# Characterization and prediction of the mechanism of action of antibiotics through NMR metabolomics

**DOI:** 10.1186/s12866-016-0696-5

**Published:** 2016-05-10

**Authors:** Verena Hoerr, Gavin E. Duggan, Lori Zbytnuik, Karen K. H. Poon, Christina Große, Ute Neugebauer, Karen Methling, Bettina Löffler, Hans J. Vogel

**Affiliations:** Institute of Medical Microbiology, Jena University Hospital, Erlanger Allee 101, D-07747 Jena, Germany; Biochemistry Research Group, Department of Biological Sciences, University of Calgary, Calgary, Canada; Department of Physiology and Pharmacology, University of Calgary, Calgary, Canada; Center for Sepsis Control and Care, Jena University Hospital, Jena, Germany; Leibniz Institute of Photonic Technology, Jena, Germany; Institute of Biochemistry, University of Greifswald, Greifswald, Germany

**Keywords:** Metabolomics, ^1^H NMR spectroscopy, Intracellular fingerprinting, Extracellular footprinting, Mode of action of antibiotics, Multivariate data analysis, Prediction of antibiotic classes

## Abstract

**Background:**

The emergence of antibiotic resistant pathogenic bacteria has reduced our ability to combat infectious diseases. At the same time the numbers of new antibiotics reaching the market have decreased. This situation has created an urgent need to discover novel antibiotic scaffolds. Recently, the application of pattern recognition techniques to identify molecular fingerprints in ‘omics’ studies, has emerged as an important tool in biomedical research and laboratory medicine to identify pathogens, to monitor therapeutic treatments or to develop drugs with improved metabolic stability, toxicological profile and efficacy. Here, we hypothesize that a combination of metabolic intracellular fingerprints and extracellular footprints would provide a more comprehensive picture about the mechanism of action of novel antibiotics in drug discovery programs.

**Results:**

In an attempt to integrate the metabolomics approach as a classification tool in the drug discovery processes, we have used quantitative ^1^H NMR spectroscopy to study the metabolic response of *Escherichia coli* cultures to different antibiotics. Within the frame of our study the effects of five different and well-known antibiotic classes on the bacterial metabolome were investigated both by intracellular fingerprint and extracellular footprint analysis. The metabolic fingerprints and footprints of bacterial cultures were affected in a distinct manner and provided complementary information regarding intracellular and extracellular targets such as protein synthesis, DNA and cell wall. While cell cultures affected by antibiotics that act on intracellular targets showed class-specific fingerprints, the metabolic footprints differed significantly only when antibiotics that target the cell wall were applied. In addition, using a training set of *E. coli* fingerprints extracted after treatment with different antibiotic classes, the mode of action of streptomycin, tetracycline and carbenicillin could be correctly predicted.

**Conclusion:**

The metabolic profiles of *E. coli* treated with antibiotics with intracellular and extracellular targets could be separated in fingerprint and footprint analysis, respectively and provided complementary information. Based on the specific fingerprints obtained for different classes of antibiotics, the mode of action of several antibiotics could be predicted. The same classification approach should be applicable to studies of other pathogenic bacteria.

## Background

The number of bacterial infectious disease outbreaks that are caused by Gram-positive and Gram-negative pathogens has increased tremendously over the past decade [1]. In particular, the increasing spread of bacterial resistance to commonly used antimicrobials has led to a significant threat in hospital as well as in community settings [2, 3]. Genetic alterations, caused by exchange of genetic material with other organisms or through mutagenesis of its own DNA, allow bacteria to overcome the action of many antibiotics [4]. Clinically important examples such as vancomycin resistance among enterococci, methicillin-resistant staphylococci, multiple resistant pneumococci and streptococci as well as multi- and pan-drug resistant Gram-negatives are increasingly being encountered in clinical settings. However, at the same time the market for research and development of new antibiotics in the pharmaceutical industry has largely evaporated [5–7].

Over the years, antibacterial drug-target interactions of the presently used antibiotics have been intensely studied, and it is well-known that these agents can inhibit various essential cellular functions such as cell wall biosynthesis, DNA supercoiling, transcription, translation, or folate biosynthesis [8, 9]. For many years, the development of resistance could be partly compensated by the synthesis of novel analogues of existing compounds. However, such chemical modifications are finite and to keep pace with the remarkable adaptability of the bacteria and to combat the prevalence of multi-drug-resistant pathogens, novel antibiotics that target distinct cellular functions are needed [10]. Consequently the focus in the drug discovery processes has moved from introducing structural variations in existing classes of antibiotics to target-based or whole cell-based high-throughput screening (HTS) of large chemical libraries in order to identify novel “hits” [6, 11, 12]. Unfortunately, these strategies have not yet led to any major successes so far [13, 14]. On the one hand, “hits” from target-based in vitro assays often turned out not to penetrate the bacterial cell and hence difficulties arose when correlating the outcome of enzymatic inhibition studies with their in vivo antimicrobial effects [11, 15]. On the other hand, in whole cell based HTS, most of the established methods for the assessment of the antimicrobial efficiency of compounds such as the broth microdilution, and the disk diffusion methods have the disadvantage of being time consuming [16–18]. Even when a HTS “hit” is identified, a series of drug development procedures is required to optimize the activity and characterize the selectivity and toxicity of novel compounds [11, 19].

In recent years further efforts have been spent using proteomic, genomic and metabolomic approaches to study the cellular processes and their responses to special antibiotic stimuli as well as potential targets [20–24]. These approaches demand a departure from the current drug discovery strategies which typically follow a linear process of identification, evaluation and refinement towards a more integrated parallel process [25]. Metabolomics in particular offers a unique strategy to detect metabolic changes that occur in an organism in response to drugs and the outcome of such studies can provide insights into their corresponding mode of action [26, 27]. In addition, different physicochemical methods such as mass spectrometry [28, 29], FT-IR spectroscopy [30], Raman microspectroscopy [31–33] and NMR spectroscopy [27, 34] have already been developed for metabolic or whole-organism profiling of the microbial response to antibiotics. In metabolomics the investigations carried out so far have focused on studying changes in the intracellular metabolism in response to antibacterial compounds. We hypothesize that a combination of intracellular fingerprinting and extracellular footprinting metabolomics approaches would give rise to a more complete mechanistic insight. To evaluate this notion, we have used ^1^H NMR to analyze metabolic changes in bacterial extracts and in the extracellular culture medium from *Escherichia coli* cultures following antibiotic treatments. Specifically, we have studied the effect of various antibiotics with different mechanisms of action to determine if the metabolomics approach can be used as a potential classification tool.

Our results show that the metabolic finger- and footprints of bacterial cultures are affected in a distinct manner by antibiotic treatments and that they provide complementary information about intra- and extracellular modes of action. Clusters of antibiotics that target intracellular processes could be separated in the fingerprint analysis. In contrast, the metabolic footprints provided distinct profiles for antibiotics that inhibit cell wall biosynthesis. Finally, we incorporated our metabolic profiling data in one descriptive model and in doing so we could predict the mode of action of several antibiotics that had not been included in the original training set.

## Results

### Antimicrobial susceptibility of *E. coli*

**Fig. 1:**
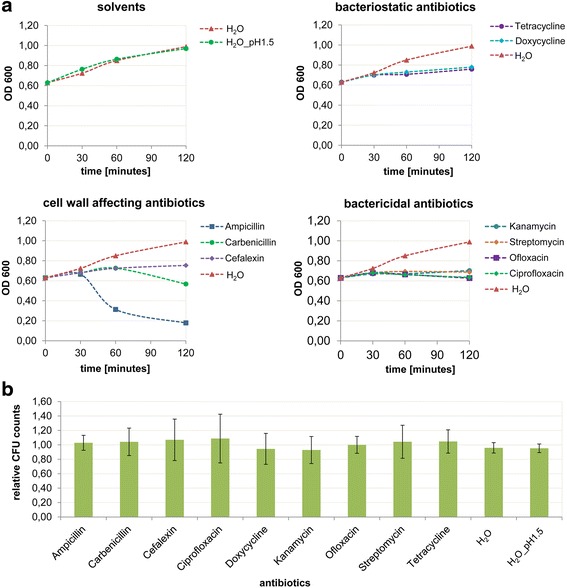
Antibiotic effect on *E. coli.* **a** OD curves and **b** CFU counts of *E. coli* cultures exposed to solvents and antibiotics with bacteriostatic, bactericidal and cell wall affecting mode of action in a concentration of 100 μM. Antibiotics affecting the cell wall resulted in significantly decreased OD values indicating that a substantial amount of cells were lysed over time course. After an incubation time of 30 min normal CFU counts were obtained for all antibiotics

In this work we have investigated the antimicrobial effect of nine antibiotics which belong to five different classes of antibiotics with different modes of action (Table 1). The MIC (minimum inhibitory concentration) values were determined for *E. coli* for all antibiotics in defined medium to characterize their antimicrobial susceptibility (Table 2). For reliable metabolite identification and quantification in the subsequent metabolomics study, a defined minimal medium was necessary to limit the presence of overlapping signals in the ^1^H NMR-spectra caused by the plethora of nutrients that are present in rich media. The different antibiotics showed MIC values ranging between 0.4 and 12.5 μM. Since the MIC value is defined as the lowest concentration of an antibiotic that inhibits the visible growth of a microorganism after overnight incubation, a concentration of 100 μM was applied for the culture experiments in the metabolomics study which is in accordance with standard guidelines for cytotoxicity assays [35, 36]. Our goal was to cause a rapid reduction in the bacterial growth rate but not to inhibit the bacterial growth completely or to induce bacterial cell death during the period of antibiotic exposure. To adjust the required incubation time, bacterial cultures were grown in the exponential growth phase to an OD600 (optical density at 600 nm) of 0.6 and aliquots were then exposed to the antibiotics in a concentration of 100 μM as well as to the corresponding solvents as controls. Except for cefalexin, ciprofloxacin and ofloxacin which were dissolved in an acidic aqueous vehicle, all antibiotics were dissolved in H_2_O (Table 2). These subcultures were tested spectroscopically after 15, 30, 60 and 120 min of incubation (Fig. 1a). Control samples reached the stationary phase of the growth curve after 120 min of incubation. Both for bacteriostatic and bactericidal antibiotics, an incubation time of 30 min already resulted in an inhibition of cell growth. Corresponding CFU (colony forming units) counts confirmed that the drug exposure was below lethal levels (Fig. 1b) and all bacterial cultures treated with antibiotics showed a survival rate of approximately 100 % compared to untreated cultures. In contrast, growth curves of bacteria exposed to bactericidal antibiotics that affect the cell wall (ampicillin, carbenicillin, and cefalexin) showed significantly decreased OD values after an incubation time of 60 min, indicating progressive cell lysis. Taken together the optimized experimental conditions to investigate the antibiotic effect on *E. coli* cultures by intra- and extracellular finger- and footprint analysis were identified after 30 min of antibiotic treatment at a concentration of 100 μM.

**Table 1:** Mode of action of different classes of antibiotics

Mode of action	Action	Antibiotics
Cell wall synthesis inhibitors: Inhibition of penicillin binding proteins (PBPs)	bactericidal	ß-lactam-antibiotics *Penicillin-derivatives:* Ampicillin, Carbenicillin *Cephalosporin-derivatives:* Cefalexin
DNA and protein synthesis inhibitors: Inhibition of topoisomerase	bactericidal	Fluoroquinolones Ofloxacin, Ciprofloxacin
Protein synthesis inhibitors: Inhibition of 30s subunit	bacteriostatic	Tetracycline derivatives Tetracycline, Doxycycline
Protein synthesis inhibitors: Inhibition of 30s subunit	bactericidal	Aminoglycosides Kanamycin, Streptomycin

**Table 2:** MIC values [μM] of different antibiotics

Antibiotics	Solvent	MIC [μM]
Ampicillin	H_2_O	2.4
Carbenicillin	H_2_O	12.5
Doxycycline	H_2_O	3.9
Kanamycin	H_2_O	6.3
Streptomycin	H_2_O	3.1
Tetracycline	H_2_O	4.7
Cefalexin	H_2_O-pH 1.5	0.6
Ciprofloxacin	H_2_O-pH 1.5	0.4
Ofloxacin	H_2_O-pH 1.5	0.4

**Fig. 2:**
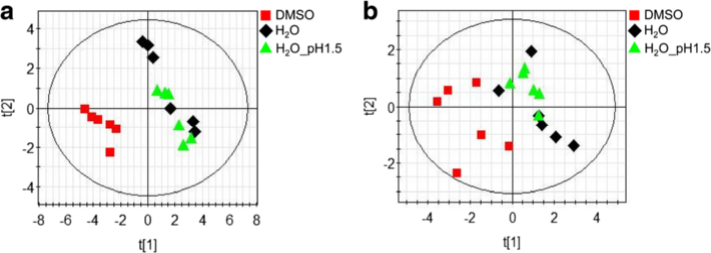
Effect of different solvents on metabolic fingerprints and footprints derived from *E. coli* cultures. **a** PLS-DA scores plot (2 components, R2 = 0.97, Q2 = 0.91) of intracellular metabolic profiles of *E. coli* cultures after incubation with different solvents (H_2_O, H_2_O_pH1.5, DMSO) in a concentration of 1 % for 30 min. **b** PLS-DA scores plot (2 components, R2 = 0.81, Q2 = 0.30) of corresponding footprints. N = 6 for each solvent

To investigate the impact of different solvents on the intra- and extracellular metabolic profiles of *E. coli*, samples of bacterial cell extracts and of the extracellular medium were prepared after 30 min of treatment with 1 % of the following commonly used solvents and solubilizers: H_2_O, an acidic aqueous solution at pH 1.5 and DMSO. It is well known that DMSO has cytotoxic properties, however, as it is often used as standard solvent for natural plant compounds in screening assays for antimicrobial or antiprotozoal characteristics, we also analyzed its influence on the metabolic fingerprint and footprint, using PLS-DA (Partial Least Squares Discriminant Analysis). Two components of the statistical analysis encapsulated 97 and 81 % of the interclass variation (R2) with a corresponding cross-validation accuracy (Q2) of 91 and 30 % for finger- and footprints, respectively. Results showed that the lower pH of the acidic aqueous solution did not affect the intra- or extracellular metabolite composition at this low concentration. However in cell extracts obtained after the treatment with 1 % DMSO, significant changes in the metabolic profiles became visible (Fig. 2a, b). Thus, only antibiotics soluble in aqueous solvents were included in our study (Table 2).

### Bacterial metabolic finger- and footprinting

**Fig. 3:**
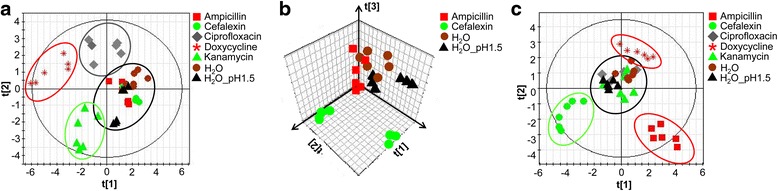
Multivariate statistical analysis of metabolic fingerprints and footprints derived from *E. coli* cultures after antibiotic treatment. **a** PLS-DA scores plot (3 components, R2 = 0.76, Q2 = 0.65) of intracellular metabolic profiles of *E. coli* cultures after incubation with antibiotics with intra- (ciprofloxacin, kanamycin, doxycycline) and extracellular (ampicillin, cefalexin) action for 30 min. **b** PLS-DA scores plot (5 components, R2 = 0.88, Q2 = 0.49) of fingerprints treated with ampicillin and cefalexin compared to controls and **c** PLS-DA scores plot of footprints corresponding to **a** (7 components, R2 = 0.72, Q2 = 0.48). *N* = 6 for each compound

**Fig. 4:**
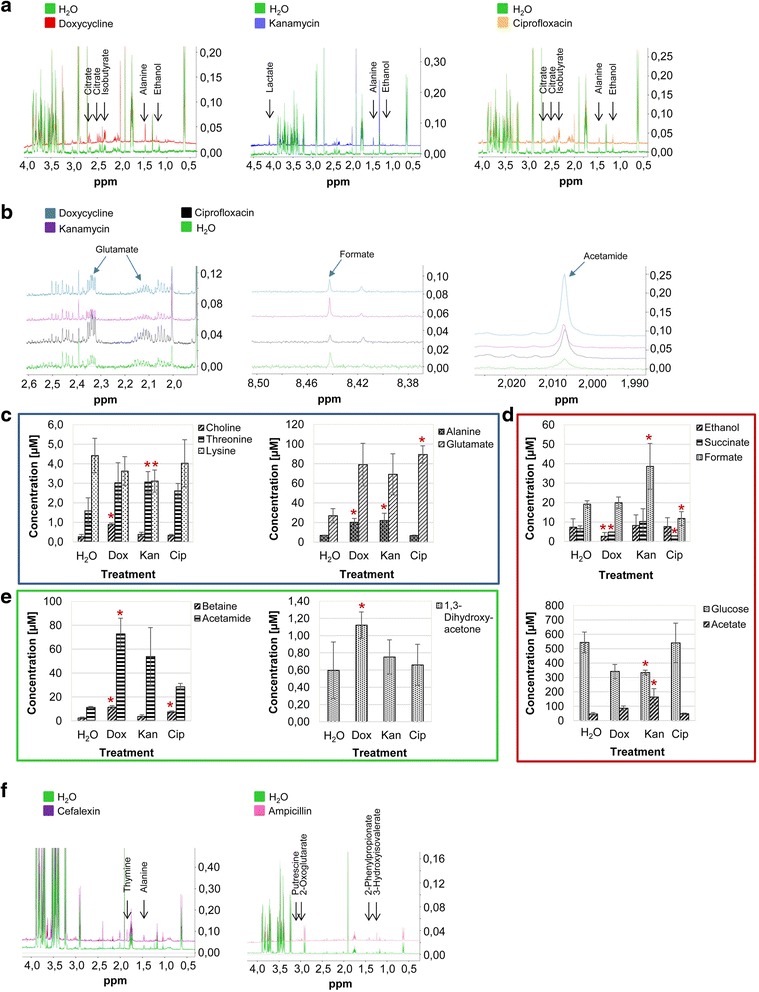
Qualitative and quantitative differences between ^1^H NMR spectra acquired from *E. coli* cultures after antibiotic treatment. **a** + **b** Noticeable metabolite changes in fingerprints after treatment with doxycycline, kanamycin and ciprofloxacin. Bar charts showing metabolite levels of important significantly increased and decreased intracellular **c** amino acids, **d** energy metabolites and **e** stress induced metabolites (* < *p* = 0.05). **f** Noticeable metabolite changes in footprints after treatment with cefalexin and ampicillin

Ampicillin, cefalexin, doxycycline, ciprofloxacin and kanamycin are five antibiotics with different modes of action (Table 1). Ampicillin and cefalexin affect cell wall biosynthesis, doxycycline and kanamycin inhibit intracellular protein synthesis while ciprofloxacin inhibits DNA synthesis. To examine the metabolic effect of these antibiotics on *E. coli*, ^1^H NMR metabolite profiles of cell extracts were compared to those of controls treated with solvent (Fig. 3a, b). PLS-DA of the metabolic fingerprint profiles (Fig. 3a) resulted in a R2 and Q2 value of 76 and 65 % respectively for the first three components. Antibiotics with intracellular and extracellular modes of action were clearly separated along the first component which explained 32 % of the total variation. Antibiotics with different intracellular targets could be clearly separated from each other in different clusters. Comparison between ^1^H NMR-spectra of cell extracts derived from different intracellular targeted antibiotics showed changed levels of alanine, glutamate, acetamide as well as of energy metabolites such as ethanol, citrate, formate and isobutyrate (Fig. 4a, b). Based on the results of the corresponding PLS-DA, significantly increased and decreased levels of amino acids (Fig. 4c), energy metabolites (Fig. 4d) and stress induced metabolites (Fig. 4e) could additionally be identified and quantified. In contrast to antibiotics with intracellular mode of action, the effects of cell wall inhibitors on the metabolic fingerprint profiles were less pronounced and antibiotic profiles were similar to those of controls (Fig. 3a; b, 5 components, R2 = 0.88, Q2 = 0.49).

To gain more insight into the metabolic effects caused by antibiotics with different antimicrobial modes of action, extracellular metabolic footprints obtained after antibiotic treatment were also statistically investigated by using a seven component PLS-DA model with a R2 and Q2 value of 72 and 48 % respectively (Fig. 3c). While the metabolomic profiles derived from antibiotics with an intracellular mode of action, such as ciprofloxacin, doxycycline and kanamycin could be differentiated from controls, the magnitude and significance of the separation was much smaller than that for the cell wall affecting clusters of ampicillin and cefalexin. For those profiles, most of the TCA (tricarboxylic acid) energy metabolites were decreased while metabolites derived from anaerobic energy pathways such as formate, acetate, and acetone were increased. Noticeable changes of specific amino acids and precursors in clusters of ampicillin and cefalexin are presented in Fig. 4f. Taken together, while metabolic fingerprint profiles extracted after treatment with antibiotics with different intracellular targets could be clearly separated from each other, metabolic footprints were necessary to differentiate cell wall affecting antibiotics.

### Cell wall affecting antibiotics analyzed by FACS and fluorescence microscopy

**Fig. 5:**
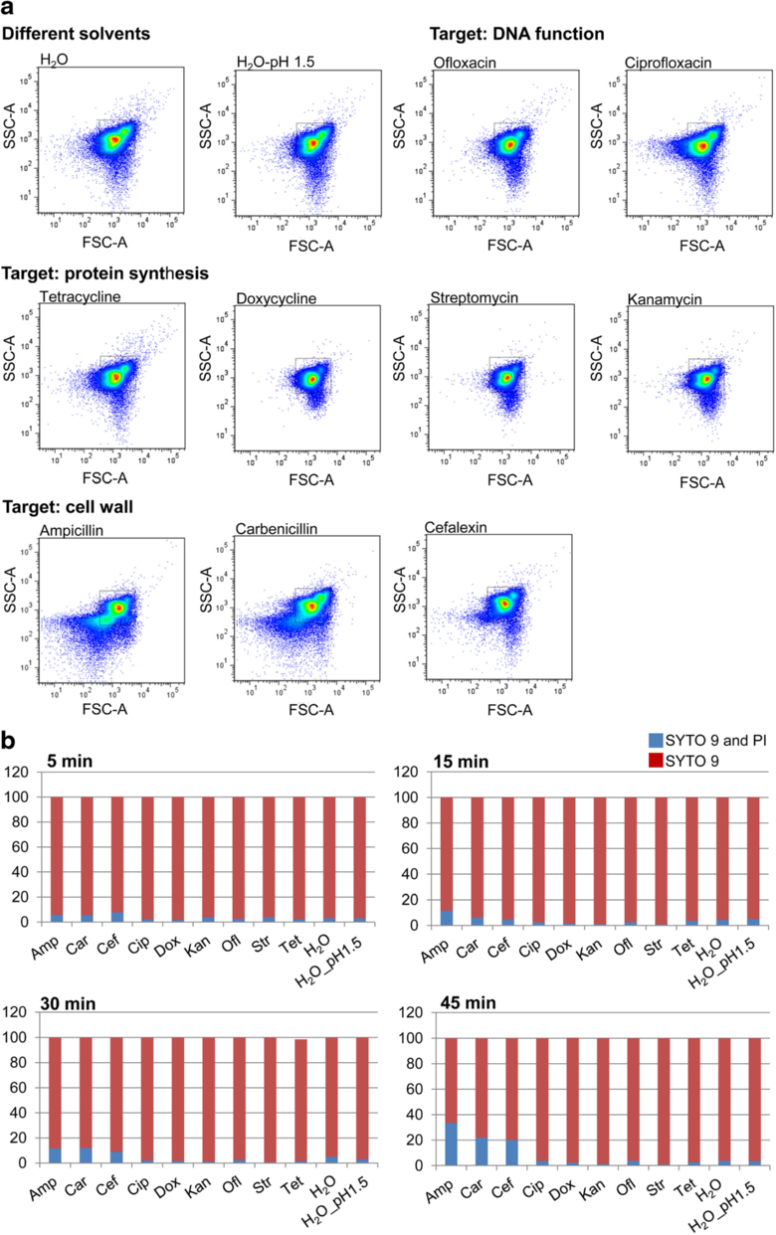
Cell wall permeability of *E. coli* after antibiotic treatment investigated by FACS. Flow cytometry measurements **a** + **b** of *E. coli* cultures stained with Syto 9 and propidium iodide (PI) after incubation with different antibiotics. **a** Forward scatter (FSC) vs Side scatter (SSC) dot plot of population of *E. coli* cells at 30 min time point. **b** Ratio of *E. coli* populations stained with propidium iodide and SYTO 9 alone post 5, 15, 30 and 45 min antibiotic treatment

**Fig. 6:**
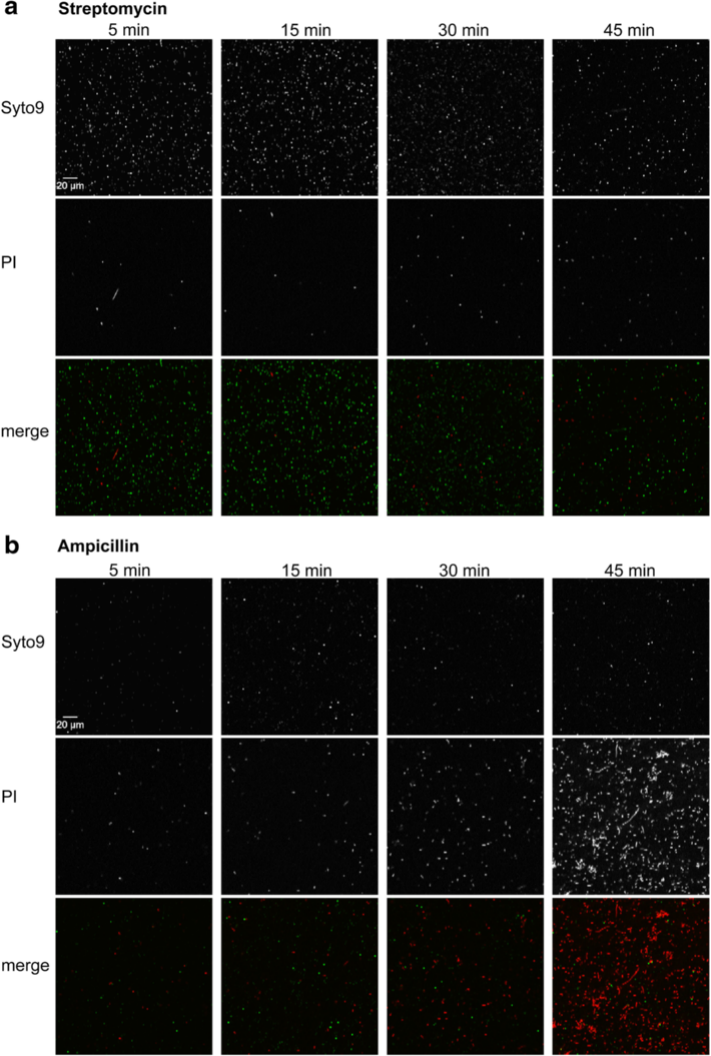
Cell wall permeability of *E. coli* after antibiotic treatment investigated by fluorescence microscopy. Fluorescence microscopy of *E. coli* cultures incubated with streptomycin **a** and ampicillin **b** for 5, 15, 30 and 45 min and stained with SYTO9 and propidium iodide (PI). SYTO9 is membrane permeant and generally labels all bacteria in a population with a green fluorescence. PI is characterized by its red fluorescence and replaces the green fluorescence in cells with reduced membrane impermeability. Images after ampicillin treatment showed increased signs of cell debris in the red fluorescence channel at 30 and 45 min time point compared to samples after streptomycin treatment

To better understand the metabolic changes, we characterized the permeability of the bacterial membrane by staining the bacteria with SYTO9 and propidium iodide (PI) after incubation with different antibiotics. SYTO9, which is membrane permeant, generally labels all bacteria in a population with a green fluorescence. In contrast, propidium iodide, characterized by its red fluorescence, is excluded by healthy cells due to membrane impermeability. In dead bacterial cells, both the displacement of SYTO9 by propidium iodide due to the stronger affinity of the latter for nucleic acids and the quenching of SYTO9 emission by fluorescence resonance energy transfer (FRET) is responsible for the replacement of the green fluorescence with the red one [37, 38]. The fluorescent bacteria were then quantified by flow cytometry (fluorescence-activated cell sorting, FACS; Fig. 5a, b) and visualized by fluorescence microscopy (Fig. 6a, b). Bacterial samples that had been incubated with cell wall affecting antibiotics such as ampicillin, carbenicillin, and cefalexin showed reduced membrane integrity, which allowed propidium iodide to cross the membrane and resulted in a significant increase in SYTO9/PI double stained population after 45 min (Fig. 5b). CFU counts in contrast were still unaffected after an antimicrobial incubation time of 30 min (Fig. 1b). A comparison of the corresponding dot plots of FSC (Forward SCatter) vs SSC (Side SCatter) for *E. coli* in the various antibiotics is shown in Fig. 5a. All cell wall affecting antibiotic samples displayed an increase in population outside the P1 gate where intact population of *E. coli* cells is located. Fluorescence microscopy confirmed the presence of a red fluorescent bacterial population already 15 min post incubation with ampicillin; these cells also showed the first signs of cell debris after an incubation time of 30 and 45 min in contrast to the control or samples treated with intracellular targeting antibiotics such as streptomycin (Fig. 6a, b).

### Prediction of antibiotic mode of action by fingerprint analysis

**Fig. 7:**
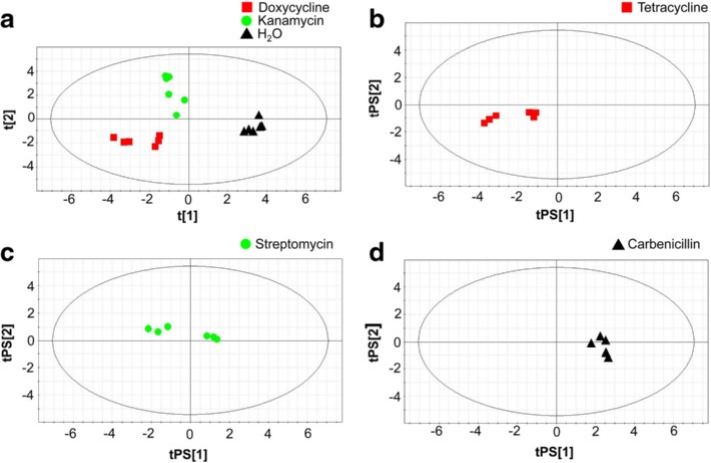
Prediction of the antibiotic mode of action. PLS-DA scores plot of fingerprints of *E. coli* cultures incubated with **a** doxycycline, kanamycin and H_2_O for 30 min (6 components, R2 = 0.99, Q2 = 0.90). Using this model as reference data set fingerprints of cultures incubated with **b** tetracycline, **c** streptomycin and **d** carbenicillin were predicted according to their mode of action. Tetracycline and doxycycline resulted in similar fingerprints as well as kanamycin and streptomycin. Carbenicillin, an antibiotic which inhibits the cell wall showed strong similarities to the control group. *N* = 6 for each compound

A six component PLS-DA model, based on the intracellular metabolite profiles obtained for doxycycline, kanamycin, and control cells, was built to predict the response of *E. coli* to treatments with streptomycin, tetracycline, and carbenicillin (Fig. 7). The model showed a R2 and Q2 value of 99 and 90 % respectively. The degree of similarity was used to predict the likelihood of each of the latter having a similar mode of action to one of the former (doxycycline and tetracycline; kanamycin and streptomycin; carbenicillin as cell wall affecting antibiotic similar to the control). The probabilities that the fingerprints of streptomycin, tetracycline and carbenicillin belong to one of the clusters of doxycycline, kanamycin or of the control is summarized in Table 3. A 88.7 % prediction was calculated that the fingerprint of streptomycin is similar to that of kanamycin. The group of tetracycline showed 76.4 % probability of being similar to the profiles of doxycycline, and the fingerprint of carbenicillin matched those of the control with a probability of 62.1 %. In each case, the highest probability was assigned to the training class with the same known mode of action or in the case of carbenicillin with that of the control. The similarity of the response can be visualized by comparing the predicted scores of the test samples to those of the training samples (Fig. 7).

**Table 3:** Prediction of mechanisms of action of tetracycline, streptomycin and carbenicillin

	Tetracycline	Streptomycin	Carbenicillin
H_2_O [%]	−2.27 ± 14.08	5.30 ± 16.65	62.13 ± 24.50
Doxycycline [%]	76.42 ± 25.84	5.96 ± 10.57	−6.29 ± 14.71
Kanamycin [%]	25.85 ± 37.39	88.74 ± 25.37	44.16 ± 11.17

Calculations based on fingerprints extracted from exposed *E. coli* cultures using a reference data set of samples treated with H_2_O, doxycycline and kanamycin

## Discussion

Increasing evidence suggests that metabolomics can be a useful tool to describe the mode of action of different antibiotics thereby driving drug discovery strategies in a more integrated parallel process of identification, evaluation and refinement [25]. Recently, it was shown that the metabolic fingerprints of bacteria affected by antibiotics with similar mode of action cluster together in statistical pattern recognition analysis [34]. In our study we investigated nine antibiotics which belong to five different antibiotic classes (Table 1) both by fingerprint and footprint analysis. Systematically, we studied the antibiotic effects on the cellular metabolism. Our results showed that metabolic fingerprints and footprints tell us different stories about antibiotics with intracellular targets and those affecting the bacterial cell wall. While antibiotics with intracellular targets showed specific fingerprints for different modes of action, cell wall affecting antibiotics induced fingerprints similar to those of control cultures and could only be separated and characterized by their corresponding footprints. Ciprofloxacin, affecting DNA supercoiling, as well as kanamycin and doxycycline, both affecting protein synthesis by different mechanisms, differed in their fingerprints significantly from control samples and cell wall inhibitors in the first and second component (Fig. 3a). Distinct amino acid levels were found after kanamycin and doxycycline treatment which might be a consequence of the inhibition of different protein synthesis pathways while ciprofloxacin mainly led to signs of general stress response showing high concentrations in betaine and glutamate [39–41] (Fig. 4c, d, e). The unique effect of antibiotics on the intracellular bacterial metabolism allowed us to distinguish between the different classes of antibiotics that act within the cell. In contrast, the corresponding footprints showed similarities in a wide range of metabolites for bactericidal antibiotics with intracellular targets but differed significantly for cell wall affecting antibiotics as well as for bacteriostatic doxycycline (Fig. 3c). All cell wall perturbing antibiotics such as ampicillin, carbenicillin and cefalexin showed enhanced membrane permeability, indicating that membrane perturbation might result in intracellular metabolite leaking. This hypothesis was confirmed by the observation of cell debris in the scatter plots (Fig. 5a) and the visualization of cells with different shape and membrane integrity by fluorescence microscopy. Since bacteria of the doxycycline treated group did not show altered membrane permeability but revealed significant changes in both finger- and footprints, it is plausible that metabolite changes in the extracellular medium are also caused by active metabolite release. The mode of action of doxycycline is based on a bacteriostatic effect which might additionally result in an active release of metabolites in the environment.

The proof of concept to use metabolomics as a powerful tool to classify antibiotics according to their different modes of action could be strengthened by predicting the class membership of antibiotics which belonged to different and known antibiotic classes, e.g. to the class of tetracyclines (tetracycline), the class of aminoglycosides (streptomycin) and the class of ß-lactam-antibiotics (carbenicillin) (Fig. 7). Nonetheless, the use of metabolomics as a classification tool for antibiotics with different modes of action is still in its infancy. Our study was restricted to antibiotics that were soluble in aqueous solutions. The high sensitivity of the method makes the use of different solvents a critical issue. Since the different chemical characteristics of novel and customary antibiotics often require the use of different solvents [36], the effect of the vehicle on the metabolomic profile might mask differences caused by different antibiotic classes (Fig. 2a, b). Even final solvent concentrations between 0.5 to 1 % in the bacterial culture which do not show any effects in standard cytotoxicity assays such as disk diffusion assays [42] can have a significant impact on the bacterial metabolism and thus make the comparison of different chemical classes difficult, as our example of DMSO showed. Also the antibiotic effect might be enhanced and might not correlate with the actual mode of action any more. One possibility would be to add DMSO in the culture medium in all experiments.

## Conclusions

In conclusion, in this pilot study we have shown that the finger- and footprint metabolite profiles of bacterial extracts from cells exposed to antibiotics can be separated based on their mode of action, and provide complementary information about intra- and extracellular processes. This orthogonal information content underscores the importance of examining two different compartments - intra- and extracellular - to obtain a more complete picture of antibiotic modes of action, and lays bare the problems which can arise when studies assume footprint profiles reflect internal changes in biochemistry. Nevertheless, in the proper context metabolic finger- and footprint profiles may be able to identify the mechanism of action of novel antibiotics. However, to better define the metabolic differences associated with different mechanisms of antibiotic action including differences between bacteriostatic and bactericidal action, a larger study will be necessary. To this end, the effect of additional intracellular acting antibiotics as well as multiple antibiotics that belong to the same class will be investigated on different clinical bacterial isolates in the future.

## Methods

### Bacteria and growth conditions

#### Bacterial strains

Experiments were performed on the Gram-negative bacterial strain of *Escherichia coli* K12 BL21 (DE3) with the genotype: F– ompT hsdSB(rB–mB–) gal dcm (DE3).

#### Cultivation

Prior to analysis, frozen stock suspensions of bacteria were cultured overnight in 5 ml of Luria-Bertani (LB) medium at 37 °C with shaking at 200 rpm. For the bacterial foot- and fingerprint analysis, 1 ml of the overnight culture was re-inoculated in 50 ml of a defined medium (900 ml H_2_0, 100 ml 10 x salt stock solution and addition of 3 g glucose, 1 g NH_4_Cl, 1 ml vitamins, 4 μl x 10 mM CuSO_4_, 4 μl x 10 mM MnCl_2_, 4 μl x 10 mM ZnSO_4_, 10 μl x 1 M CaCl_2_, 2 ml x 1 mM FeSO_4_, 4 ml x 1 M MgSO_4_). 1 l of the 10 x salt solution contained: 60 g Na_2_HPO_4_, 30 g KH_2_PO_4_ and 5 g NaCl. Subsequently the culture was grown up to an OD600 (optical density at 600 nm) of 0.6 and was used for antibiotic treatment and cytotoxicity assays.

### Antibiotics and stock solutions

Antibiotics: ampicillin sodium, carbenicillin disodium, tetracycline, doxycycline hyclate, kanamycin sulfate, streptomycin sulfate, ofloxacin, cefalexin, ciprofloxacine were purchased from Sigma Aldrich, Oakville, Ontario, Canada.

Antibiotic stock solutions: All antibiotics were dissolved to yield a stock solution of 10 mM. Ampicillin, carbenicillin, tetracycline, doxycycline, kanamycin, streptomycin were dissolved in distilled H_2_O (pH = 6.5); ofloxacin, ciprofloxacin and cefalexin in an aqueous solution at a pH of 1.5 using HCl.

### Cytotoxicity assays

#### MIC

The assay standardized by the DIN 58940–8 guideline [35] was performed under modified conditions. In microtiter plates, 100 μL of the exponential phase cultures adjusted to a cell density of 2 × 10^7^ cells/mL (1:10 dilution of a bacterial culture at an OD600 of 0.6) were incubated with 100 μL of 2-fold serially diluted antimicrobial agents in the defined minimal culture medium at 37 °C for 24 h. The dilution series was in the range of 100 μM and 100 pM. The MIC, determined by microplate reader at 550 nm (*n* = 3), was regarded as the lowest antibiotic concentration where growth of the bacteria after 24 h incubation was inhibited.

#### OD600

50 ml of *E. coli* cultures at an OD600 of 0.6 were exposed to different antibiotics in a concentration of 100 μM. After 0, 30, 60 and 120 min of incubation an aliquote was taken and the corresponding OD value was measured at 600 nm (*n* = 3).

#### *CFU* counts

1 ml of the 50 ml bacterial culture at an OD600 of 0.6 was aliquoted into 15 ml culture tubes and exposed to different antibiotics in a concentration of 100 μM for 5, 15 and 30 min.

The corresponding viable cell concentration after treatment was determined by counting the CFUs. Therefore, a bacterial sample was serially diluted and a known volume was plated onto LB agar. The cell concentration resulted from counting the colonies that had formed and was calculated relatively to a control sample without antibiotic treatment (*n* = 3).

#### FACS and fluorescence microscopy

1 ml of the 50 ml bacterial culture at OD600 of 0.6 was aliquoted into 15 ml culture tubes and exposed to different antibiotics at a concentration of 100 μM for 5, 15, 30 and 45 min. To each time point, the cell aliquot was washed with PBS, stained with SYTO9 and propidium iodide life dead marker and analyzed by FACS or fluorescence microscopy (*n* = 2).

#### Fluorescent staining

To 1 ml of the bacterial aliquot suspended in PBS an amount of 1 μl of both 0.33 mM SYTO9 and 10 mM propidium iodide was added for fluorescent labeling. After an incubation time of 15 min in the dark, the samples were investigated by FACS or fluorescence microscopy.

### Flow cytometry analysis

Flow cytometry experiments were conducted on a BD Biosciences LSRII cytometer controlled by BD FACSDiva software. SYTO9 excitation was performed with a 488 nm laser and fluorescence emission detected with a 525/50 nm band pass filter. Green laser at 532 nm was used to excite propidium iodide and fluorescence emission was captured with a 610/20 nm band pass filter. Data analysis was done using FACSDiva software, Flowjo (Tree Star, Inc).

### Fluorescence microscopy

Fluorescence microscopy was performed using an LSM 780 (Zeiss, Jena, Germany) confocal laser scanning microscope equipped with a 20x/0.8 Plan-Apochromate Objective and a photomultiplier tube as detector. For excitation, the following lasers were used: an argon laser with 488 nm wavelength and power of 0.05 mW for excitation of SYTO9 fluorescence and a diode-pumped solid-state laser with 561 nm wavelength and power of 0.2 mW for excitation of PI fluorescence. Fluorescence emission was detected in the range of 493 to 592 nm with a detector gain of 630 for SYTO9 and in the range of 566 to 718 nm with a detector gain of 650 for PI. The Pinhole diameter was set to 32 μm for SYTO9 and to 38 μm for PI detection, respectively. Scans were recorded subsequently as two separate tracks to avoid cross-talk of laser excitation. Scanning time was kept short by using a pixel dwell time of 1.58 μs, and 8-bit images of 512 × 512 pixel were generated. For figure preparation and visualisation, the images were cropped to 256 × 256 pixel and the contrast and brightness for SYTO9 images was slightly increased.

### Intracellular metabolite extraction by cold methanol (fingerprinting)

After incubating 50 ml *E. coli* cultures at OD600 = 0.6 with different antibiotics at a concentration of 100 μM for 30 min, the total amount of 50 ml were collected on separate 0.22 μm filters [43]. Immediately after the disappearance of the culture medium the unwashed filters were transferred to liquid nitrogen. Subsequently, each filter was crushed into separate conical tubes and was suspended in 5 ml of 100 % cold methanol which was cooled to below −40 °C in a dry-ice-ethanol bath. Three freeze-thaw cycles were applied to break the cell wall and to release the intracellular metabolites. The lysates were centrifuged at 4000 g for 15 min at 4 °C and the supernatants were transferred into a clean tube. The metabolites were purified by adding 5 ml of water and 10 ml of chloroform and shaking for 5 min. The water–methanol phase contained the metabolites and was separated from the lipophilic chloroform-phase by centrifugation for 15 min at 4000 g. The metabolite solution was transferred to a clean tube and two times one milliliter was aliquoted into 1.5 ml-tubes. The samples were dried down in a speed-vacuum evaporator, resuspended in 500 μl of D_2_O and stored at −20 °C until NMR measurement.

### Cultural supernatant (footprinting)

Samples for footprinting were prepared simultaneously to intracellular metabolite extraction. After separating bacterial cells by filtration the filtrate was stored at −20 °C until NMR measurements.

### NMR sample preparation

Samples for footprint and fingerprint-analysis were filtered in 3-kDa-cutoff spin columns (3 K Nanosept: Omega; VWR, Edmonton, Alberta, Canada) and filtered protein was rinsed using additional 100 μl of D_2_O. Prior to use, filters had been washed 5 times with water and then D_2_O to remove the glycerol used to preserve the columns. A final sample volume of 450 μl was transferred to clean microfuge tubes. Samples were brought to a total volume of 650 μl by addition of D_2_O, 40 μl of sodium azide (1 M NaN_3_ solution) 140 μl of phosphate buffer (500 mM NaH_2_PO buffer solution at pH 7.0) containing 2.5 mM of 2,2-dimethyl-2-silapentane-5-sulfonate (DSS) as internal standard and the pH was adjusted to 7.00 ± 0.05.

### NMR acquisition

All experiments were acquired on a Bruker AVANCE 600 spectrometer equipped with a 5 mm TXI probe (Bruker, Milton, ON, Canada) at 298 K. The 1D ^1^H NMR spectra were acquired using a standard Bruker 1D nuclear Overhauser enhancement spectroscopy (NOESY)-presaturation pulse sequence (noesypr1d) [44, 45] in which the residual water peak was irradiated during the relaxation delay of 1.0 s and during the mixing time of 100 ms. For an overall recycle time of 5 s an acquisition time of 2 s and an initial relaxation delay of 3 s was used. A total of 1024 scans were collected for samples of culture experiments. All spectra were acquired zero filled and Fourier transformed to 64 K points. The obtained spectra were manually corrected for phase and baseline distortions within Topspin (Bruker Biospin, Ltd.) and were referenced to the DSS resonance at 0.0 ppm. Additionally, two-dimensional NMR experiments such as total correlation spectroscopy (2D ^1^H-^13^C TOCSY) and heteronuclear single quantum coherence spectroscopy (2D ^1^H-^13^C HSQC) were performed for chemical shift assignments and verification.

### Targeted metabolite profiling

1D ^1^H NMR spectra were imported into Chenomx NMR Suite version 4.6 (Chenomx Inc., Edmonton, AB, Canada) for metabolite identification and quantification by targeted metabolite profiling analysis in the Profiler module which is linked to a library representing over 260 metabolite entries [46]. To determine the concentration of individual compounds the concentration of DSS was used as reference. Each compound concentration was then normalized to the sum of all concentrations [47]. These normalized data were then used for multivariate data analysis. Altogether 23 metabolites were profiled in bacterial fingerprints and 20 metabolites were identified in footprints.

### Multivariate data analysis

For footprint and fingerprint analysis, 6 samples were prepared respectively using three biological replicates and two samples per replicate. Chemometric analysis was performed using SIMCA-P version 11.5. A supervised projection technique (partial least-squares discriminant analysis, PLS-DA) was applied where permutation validation suggested that a reliable model could be built. Models were optimized using leave-one-out cross-validation, comparing the Q2 cross-validation estimate of accuracy with R2Y training estimates of predictive power. A threshold improvement of 5 % in the Q2 metric was used as a cutoff for significant components.

To reveal differences in the metabolomic profiles specific to the antibiotic treatments, supervised PLS-DA modeling was applied with binary contrast variables to indicate class-membership for each treatment.

### Prediction of antibiotic class membership

Because the ability of trained PLS-DA models to classify novel antibiotics is of primary importance, a prediction methodology was applied to three antibiotics (tetracycline, streptomycin and carbenicillin) previously unseen in the training process but whose mode of action was known a priori from the literature (Table 1). A model, trained to differentiate two antibiotics with different modes of action (doxycycline, kanamycin) from control samples diluted with H_2_O, was used to predict scores for the three alternate test treatments. The distribution of predicted scores for test samples was compared to that of each treatment in the training dataset, for which SIMCA calculated membership probabilities. The training class with the highest similarity, and hence probability of membership, was taken to be the predicted mode of action for each test treatment, and then compared to the known expectation.

### Ethics approval and consent to participate

Not applicable.

### Consent for publication

Not applicable.

### Availability of data and materials

The data sets supporting the results of this article are included within the article.

## Abbreviations

CFU: colony forming units
DSS: 2,2-dimethyl-2-silapentane-5-sulfonate
FACS: fluorescence-activated cell sorting
FRET: fluorescence resonance energy transfer
FSC: Forward Scatter
FT-IR: fourier transform infrared
HTS: high-throughput screening
MIC: minimum inhibitory concentration
NMR: nuclear magnetic resonance
OD600: optical density at 600 nm
PI: propidium iodide
PLS-DA: Partial Least Squares Discriminant Analysis
Q2: cross-validation accuracy
R2: interclass variation
SSC: Side Scatter
TCA: tricarboxylic acid
